# Increasing Incidence of Listeriosis in France and Other European Countries

**DOI:** 10.3201/eid1405.071395

**Published:** 2008-05

**Authors:** Véronique Goulet, Craig Hedberg, Alban Le Monnier, Henriette de Valk

**Affiliations:** * Institut de Veille Sanitaire, Saint Maurice, France; † University of Minnesota School of Public Health, Minneapolis, Minnesota, USA; ‡ Institut Pasteur, Paris, France

## Abstract

From 1999 through 2005, the incidence of listeriosis in France declined from 4.5 to 3.5 cases/million persons. In 2006, it increased to 4.7 cases/million persons. Extensive epidemiologic investigations of clusters in France have ruled out the occurrence of large foodborne disease outbreaks. In addition, no increase has occurred in pregnancy-associated cases or among persons <60 years of age who have no underlying disease. Increases have occurred mainly among persons >60 years of age and appear to be most pronounced for persons >70 years of age. In 8 other European countries, the incidence of listeriosis has increased, or remained relatively high, since 2000. As in France, these increases cannot be attributed to foodborne outbreaks, and no increase has been observed in pregnancy-associated cases. European countries appear to be experiencing an increased incidence of listeriosis among persons >60 years of age. The cause of this selective increased incidence is unknown.

## Abstract 2

The cause of an increase among persons >60 years of age in France is unknown.

Surveillance methods for listeriosis vary across Europe (*1*). Reported incidence of listeriosis ranged from 0 to 7.5 cases/million persons in 2002; the highest rates were reported from countries with statutory reporting of cases and surveillance through a national reference laboratory (*1*). In recent years, interest in developing a European surveillance network for listeriosis has led to enhanced surveillance activities in several countries and has generally heightened awareness of the public health importance of *Listeria monocytogenes*. The epidemiologic picture that has emerged from recent national surveillance reports suggests that rates of listeriosis across Europe have been increasing or have remained stable at relatively high levels (*2*). In contrast, the incidence of listeriosis in France declined from 4.5 cases/million persons during 1999–2000 to ≈3.5 cases/million during 2001–2003 (*3*).

In 2006, however, France reported an increase in the incidence of listeriosis. This increase appears to share many of the epidemiologic features of recent increases in other European countries. We describe the emerging epidemiology of listeriosis in France and discuss it in the context of recent increases in other European countries.

## Methods

### Data Collection from France

Surveillance of human listeriosis in France has been conducted with consistent methods since 1999 (*4*). These methods include mandatory reporting of cases (monitored by Institut de Veille Sanitaire [InVS]) and voluntary submission of *L. monocytogenes* strains to the National Reference Center for Listeria, Institut Pasteur, Paris (NRC). By using these complementary approaches, information about clinical data, demographic data, and food consumption can be collected for each patient. In addition, temporal trends in disease occurrence can be tracked, and possible common sources of exposure can be identified among cases in clusters detected by NRC.

### Definitions

A case of listeriosis was defined by isolation of *L. monocytogenes* from a patient with a clinically compatible illness. A case is considered maternal/neonatal when it involves a pregnant woman, a miscarriage, a stillbirth, or a newborn <1 month of age. *L. monocytogenes* isolated from both a pregnant woman and her newborn child is counted as a single case. If a case fits none of these groups, it is considered not maternal/neonatal. Patients are considered to be at risk if they have an underlying pathologic condition that weakens their immune system, such as cancer, blood malignancy, organ transplant, chronic hemodialysis, liver failure, diabetes, HIV, or treatment with cytolytic or corticosteroid immunosuppressants. A cluster was defined as the occurrence of at least 3 listeriosis cases that involved strains of the same pulsovar over a period of 1) 14 weeks during January 2000–July 2006 or 2) 6 weeks after July 2006. Cases not belonging to a cluster were defined as sporadic cases.

Mandatory reporting includes submission of case-related information on a standard report form. This form includes information such as the patient’s district of residence, patient’s age, the clinical form of disease, the possible existence of an underlying illness, and whether the patient died before follow-up. A standard questionnaire is administered in person or by telephone to ascertain food items consumed in the 2 months before onset of illness, including food items previously identified as vehicles in outbreaks and foods that have been previously found to be contaminated by *L. monocytogenes* and typically consumed uncooked. Answers are entered into a food-exposure database.

### Analysis of Strains by NRC

*Listeria* isolates from patients and foods referred to NRC were confirmed with API Listeria (bioMérieux, Marcy l’Etoile, France) and serotyped by the classic technique until January 2005 (*5*) and by multiplex PCR (*6*) since February 2005. According to our experience, PCR group fully corresponds to the 4 major serovars that cause human disease. Ongoing subtyping was conducted by DNA macrorestriction profiles analysis (pulsed-field gel electrophoresis) according to standard protocols (*7*). Isolates with indistinguishable Apa1 and Asc1 DNA macrorestriction profiles, first based on visual comparison of banding patterns (since 2006 by using BioNumerics 4.5 software [Applied Maths Saint-Martens-Latem, Belgium]), were considered to be the same pulsovar. Detected clusters were reported to InVS for investigation (*3*).

### Data Analysis

Data analysis was performed with Epi-Info version 6.04 (Centers for Diseases Control and Prevention, Atlanta, GA, USA). Incidence rate ratios (RRs) were calculated with Stata version 8.2 (StatCorp, College Station, TX, USA). For the data from France, we compared incidence rates in 2006 and 2007 with the mean incidence over the precedent 5-year period (2001–2005). To estimate the incidence data for 2007 by extrapolation of the incidence observed from January to June 2007, we multiplied the incidence from January to June by a factor of 1.2, which represents the mean annual multiplier (annual cases/cases January–June) observed from 2001 through 2006.

#### Data from Europe

We conducted an Internet search for surveillance data from the institutes in charge of infectious disease surveillance in Western European countries. We also reviewed articles published on listeriosis trends in European countries with data for 2000–2006.

## Results

### Data from France

#### Epidemiologic Characteristics

The annual incidence of listeriosis in France decreased in 2001 (Table 1) and stabilized until 2005 at ≈3.5 cases/million persons. In 2006, the incidence increased to 4.7 cases/million persons. In 2007, 159 cases were reported from January through June, which corresponds to an estimated incidence of 5.6 cases/million persons. For the 6-month period from January through June, the incidence of listeriosis increased by 46% in 2006 and 2007 compared with incidence during 2001–2005 (RR 1.4; 95% confidence interval [CI] 1.2–1.6; p<0.001).

**Table 1:** Incidence and characteristics of cases of listeriosis by year, France

Characteristic	1999	2000	2001	2002	2003	2004	2005	2006
No. cases reported	269	263	188	220	209	236	221	290
Incidence/1 million inhabitants	4.5	4.4	3.1	3.6	3.4	3.8	3.5	4.6
Clinical form								
Maternal/neonatal	67	64	44	55	47	49	39	36
Not maternal/neonatal	202	199	144	165	162	187	182	254
Bacteriema	122	110	85	89	100	124	115	171
Central nervous system infection	65	73	51	67	54	53	60	65
Focal infection	15	16	8	9	8	10	7	18

**Figure 1:**
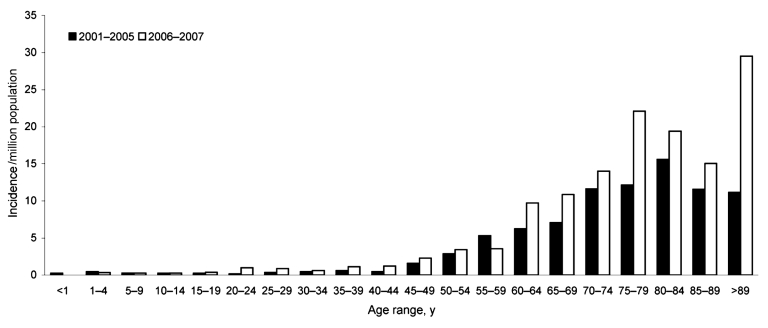
Trends of non–maternal/neonatal listeriosis by age, France, January 1, 2006–June 30, 2007, versus January 1, 2001–December 31, 2005.

The increased incidence of listeriosis in 2006–2007 over that of 2001–2005 was mainly due to a rise in cases in persons >60 years of age (+51%; RR 1.6; 95% CI 1.4–1.8; p<0.001) and was most pronounced in those >75 years of age (+58%; RR 1.7; 95% CI 1.4–2.1; p<0.001) (Figure 1). This increase was observed in persons >60 years of age, regardless of whether they had a recognized underlying condition. The mortality rates among these cases did not increase. From 2001–2005 to 2006 and 2007, there was a larger overall increase in bacteremia cases (+67%) than in central nervous system cases (+35%).

The regional distribution of cases did not differ significantly from 1 region to another during 2006 and 2007 versus during 2001–2005. The increase was similar for sporadic cases and cluster-associated cases (Table 2). A seasonal effect, with an increase of cases during summer, was observed in 2006, as in preceding years. However, information about food consumption of patients >60 years of age showed that a decrease in consumption of foods considered to pose a risk for listeriosis occurred in 2006–2007 compared with 2001–2005.

**Table 2:** Clusters of listeriosis, cluster-associated cases, and sporadic cases, France, 2000–2006

Characteristic	2000	2001	2002	2003	2004	2005	2006
No. clusters detected	9	4	10	11	13	11	11
No. cases belonging to a cluster	53	21	70	78	88	65	98
No. sporadic cases	210	167	150	131	148	156	192

**Figure 2:**
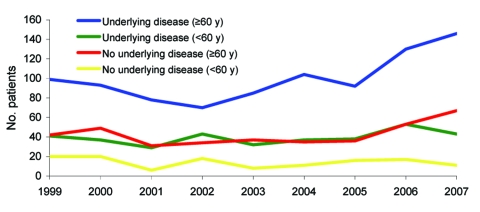
Trends of non–maternal/neonatal listeriosis by presence of underlying disease and age of patients, France, January 1, 1999–June 30, 2007. (Data for 2007 are estimated.)

**Figure 3:**
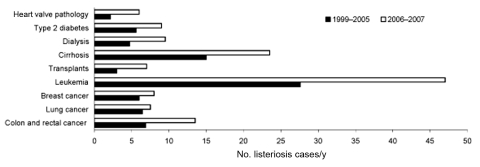
Trends (no. cases of listeriosis/y) by underlying medical condition, France, January 1, 1999–June 30, 2007.

For persons <60 years of age, a 32% increase was observed only in patients with an underlying condition that increased their risk for listeriosis (Figure 2), particularly patients with leukemia (Figure 3). The incidence of maternal/neonatal cases of listeriosis has decreased continuously from 1999 to 2006, with the same trend in the first half of 2007. In 2006, maternal/neonatal cases represented 12% of cases.

#### Strains Analysis

In 2006, NRC received 280 *L. monocytogenes* strains, which accounted for 96.5% of cases reported to InVS. The distribution of strains by serovar/PCR group did not change from 2001 through 2006 (Table 3). The most common serovar was 4b, which accounted for half of all strains. Analysis of PCR group for strains received during the first 6 months in 2007 shows the same results in terms of distribution. As in previous years (*3*), serovar 4b was predominant among maternal/neonatal cases and central nervous system infection cases and more frequent than among bacteremia cases (Table 4).

**Table 3:** Distribution of strains of *Listeria monocytogenes* isolated from human case-patients by serovar, by year

Characteristic	1999	2000	2001	2002	2003	2004	2005	2006	2007*
No. of strains	240	222	186	202	197	233	212	280	151
Serovar 1/2a, %	27	33	33	22	26	30	24	29	27
Serovar 1/2b, %	20	16	22	18	22	11	17	17	15
Serovar 1/2c, %	5	3	3	4	5	4	3	4	4
Serovar 4b, %	48	48	42	55	47	55	56	50	54
Other serovar, %	<1	0	0	<1	0	0	0	<1	<1

*Through Jun 30.

**Table 4:** Distribution of *Listeria monocytogenes* strains isolated in 2006 from human case-patients, by serovar and clinical forms

Characteristic	No. (%) 1/2a	No. (%) 1/2b	No. (%) 1/2c	No. (%) 4b	No. (%) other	Total no.
Not maternal/neonatal	76 (31)	43 (18)	11 (4)	115 (47)	1 (<1)	246
Central nervous system infection	13 (24)	9 (16)	2 (4)	30 (54)	1 (2)	55
Bacteremia	54 (31)	31 (18)	9 (5)	81 (46)	0 (0)	175
Focal infections	9 (56)	3 (19)	0 (0)	4 (25)	0 (0)	16
Maternal/neonatal	4 (12)	6 (18)	0 (0)	24 (70)	0 (0)	34
Total	80 (29)	49 (17)	11 (4)	139 (50)	1 (<1)	280

#### Cluster Detection and Investigations

In 2006, 102 pulsovars were identified among the 280 isolates, with 1–30 isolates/pulsovar (Table 5). Pulsed-field gel electrophoresis analysis identified 11 clusters: 9 involving strains of PCR group IVb and 2 of PCR group IIa (Table 5). Results of intensive epidemiologic investigation did not show any cluster suggestive of common-source outbreaks. In 1 cluster involving 14 cases, an *L. monocytogenes* strain of the case-associated pulsovar was identified in a sheep raw milk cheese that had been consumed by 3 patients but not by the other patients in the cluster. The proportion of strains related to a cluster in 2006 (34%) was similar to that in 2003–2005 (35%) (Table 2).

**Table 5:** Description of the diversity, according to serovar, of *Listeria monocytogenes* strains isolated in 2006 for strains belonging to serovars 1/2a, 1/2b, 1/2c, and 4b

Characteristic	1/2a	1/2b	1/2c	4b	Total
No. *L. monocytogenes* strains	80	49	11	139	279
No. pulsotype	42	26	6	28	102
No. pulsotype found once	32	18	4	13	67
Range of frequency for each pulsotype, min–max	1–17	1–10	1–5	1–30	1–30
No. clusters	2	0	0	9	11
Index of diversity	0.931	0.923	0.727	0.893	0.968

### Data from Europe

Complete annual incidence data from 2000 through 2006 were available for 5 European countries (Table 6). In 2000, the median incidence was 4.7 cases/million persons (range 1.9–7.5 cases/million persons); in 2006, it was 6.3 cases/million persons (range 3.5–10.3 cases/million persons) in these countries. Increases were observed for Belgium, Denmark, England, Wales, and Finland. In Sweden, the incidence decreased during 2000–2006. However, the incidence in Sweden was already high in 2000–2001 (5.9–7.5 cases/million persons). Incidence data for at least 5 years during this period were also available for Germany, the Netherlands, and Switzerland. They all observed increases over this period.

**Table 6:** Annual incidence of listeriosis in 8 European countries

Country	Source	2000	2001	2002	2003	2004	2005	2006
Belgium	Scientific Institute of Public Health*	4.7	5.6	4.3	7.3	8.6	5.9	6.4†
Denmark	Statens Serum Institut‡	7.5	7.1	5.2	5.4	7.6	8.5	10.3†
England-Wales	Health Protection Agency§	1.9	2.8	2.6	4.5	4.0	3.5	3.5
Finland	National Public Health Institute¶	3.5	5.4	3.8	7.9	6.7	6.8	8.5
Germany	Robert Koch Institute#		2.6	2.9	3.1†	3.6†	6.2	6.2†
Netherlands	National Institute of Public Health**			2	3	3	5.6††	3.9†
Sweden	Swedish Institute for Infectious Disease Control‡‡	5.9	7.5	4.4	5.3	4.7	4.4	4.6
Switzerland	Office Fédéral de la Santé Publique§§		5.1	3.8	6.1	7.8	9.8	9.1

*www.iph.fgov.be/bacterio/iframes/rapports/2005/Listeria_2005_FR_web.pdf. †www.efsa.europa.eu/EFSA/efsa_locale-178620753812_1178671312912.htm. ‡www.ssi.dk/sw44830.asp. §www.hpa.org.uk/infections/topics_az/listeria/data_ew.htm. ¶www3.ktl.fi/stat. #www.eurosurveillance.org/em/v11n06/1106-124.asp. **www.rivm.nl/infectieziektenbulletin/bul1712/art_listeria.html. ††Introduction of active surveillance. ‡‡www.smittskyddsinstitutet.se/in-english/statistics/listeriosis. §§www.bag.admin.ch/k_m_meldesystem/00733/00804.

In England and Wales, Gillespie et al. compared 2 periods: 1990–2000 and 2001–2004 (*8*). They showed that the increase resulted from a rise in sporadic cases, predominantly in patients >60 years of age. The increase was independent of sex, ethnicity, or economic status and occurred in most regions of England and Wales. The proportion of serovar 4b and 1/2 isolates and the proportion of persons with underlying illness did not change during this period. The proportion of bacteremic patients >60 years of age increased significantly during 2001–2004 versus 1990–2000 (85% vs. 76%); after 2000, the risk among persons >70 years of age was higher than that for persons 60–69 years of age. During the periods compared by Gillespie et al., the incidence of maternal/neonatal cases did not change.

In Germany, surveillance data showed a continuous increase of cases since 2001, when the national reporting system was introduced. A particularly steep increase was observed in 2005, when the number of cases increased by 72%. Analyzing this increase, Koch and Stark reported that temporal and spatial distribution of cases did not show any clusters suggestive of local outbreaks (*9*). From 2001 through 2005, the number of cases in those >60 years of age increased by a factor of 2.6. Among persons >80 years of age, almost 4 times as many cases were reported in 2005 as in 2001. In the same period, the annual number of maternal/neonatal cases did not change.

In the Netherlands, until 2005, information was based on data from 15 regional public health laboratories that cover an estimated 44% of the population and from the Netherlands Reference Laboratory for Bacterial Meningitis, which receives isolates from patients with meningitis or septicemia. According to these data, the annual incidence of listeriosis had been stable until 2002 at ≈2 cases/million persons and has increased since 2003 to ≈3 cases/million persons (*10*). Since 2005, an active surveillance that involved all laboratories has been implemented. Cases reported by Doorduyn et al. in 2005 (*10*) corresponded to an incidence of 5.6/million persons (Table 3). Although this increase may have resulted from the strengthening of listeriosis surveillance, the authors do not rule out a genuine recent increase in the number of cases.

In Switzerland, an increase has been observed since 2004. In 2005, an outbreak involving 12 cases (16% of cases reported in 2005) was linked to consumption of a cheese, accounting only for a small part of the upsurge (*11*). In 2006, the incidence remained high (9.1/million persons), although no common-source outbreak was identified.

In Denmark, an increase since 2004 was caused by various subtypes of *L. monocytogenes;* this increase was likely not the consequence of a single outbreak (*12*). This increase involved septicemia cases but not meningitis cases. A further increase was observed in 2006, leading to an incidence of 10.3 cases/million persons.

In Finland, an increase has been observed since 2003. Clusters are detected by routine serogenotyping of strains. In 2003–2004, 2 clusters with 7 cases each were investigated. In 1 cluster, food histories implicated cold fish products (*13*). Clinical and demographic characteristics of cases occurring in 2003–2004 and those occurring in 1999–2000 did not differ.

In Belgium, the incidence has increased since 2003 and reached a peak of 8.6 cases/million persons in 2004 (*14*). The large increase in 2004 was mainly caused by increasing cases in the Flemish community, and the proportion of isolates of serovar 1/2 was unusually high (68%). Therefore, the occurrence of a common-source outbreak in 2004 cannot be ruled out.

## Discussion

Surveillance data in France show an upsurge in human listeriosis cases in 2006; this increase was confirmed in January–June 2007 such that the annual incidence is now at its highest level since 1998, when mandatory reporting was introduced. This upsurge is due to an increase among persons >60 years of age who were not pregnant and among persons <60 years of age who had a predisposing medical condition.

The methods and conditions of listeriosis surveillance in France have remained unchanged since 1998, and the sensitivity of the mandatory reporting system is high (*15*). In the 1990s, large outbreaks of listeriosis increased awareness among physicians and microbiologists. Because no large outbreaks occurred since 2000, this upsurge is most likely not the result of better reporting or raised diagnostic awareness. The increase of bacteremic cases could be an artifact caused by higher hospitalization rates among the elderly, increased frequency of performing blood cultures, or increased sensitivity of the blood-culturing systems. However, from 2005 through 2006, for persons >60 years of age, the incidence of listeriosis jumped 39%, the hospitalization rates increased <1%, and data from the national insurance scheme showed a 20% reduction in blood cultures. Because instrumented blood culture systems have been used in hospitals for several years with no demonstrated improvement in the sensitivity of detecting *L*. *monocytogenes*, the increase of bacteremic infections does not appear to be due to diagnostic practices.

In 2006, the proportion of cases related to clusters remained stable; the clusters did not account for the upsurge in incidence. Also, multiple *L. monocytogenes* strains were responsible for the increase in cases. Because of the above reasons, the increase in incidence in France is unlikely to be due to a common-source outbreak.

In several European countries, similar trends of increasing listeriosis case numbers have been observed. For countries with a long history of listeriosis surveillance, such as England, Wales, Switzerland, and Denmark, the observed upsurge is likely genuine. But even for countries with recently introduced or strengthened listeriosis surveillance systems, such as the Netherlands and Germany, the observed upsurge is not attributed to a surveillance artifact. The upsurge is due to an increase in cases in the same patient groups.

In France, Germany, England, and Wales, the increased incidence occurred predominantly in patients >60 years of age. The number of maternal/neonatal cases is declining in all countries. Clusters suspected or confirmed to represent common-source outbreaks contributed to the increased incidence in some countries, such as in Switzerland in 2005, in northeast England in 2003, in Finland in 2003–2004, and possibly the Netherlands and Belgium in 2004. However, in none of these countries did these clusters account for the upsurge in incidence.

Many of these same epidemiologic features may also be occurring in the United States, where a decline in the incidence of listeriosis from 1996 to 2003 was reported on FoodNet websites (*16*). The incidence of listeriosis declined from 4.1 to 2.3/million persons from 1996 to 2003; the percentage of maternal/neonatal cases dropped from 15% to 11% during this period. After dropping to a record low incidence of 2.7 cases/million persons in 2004, the incidence of listeriosis cases reported on FoodNet websites increased to 3.0 cases/million persons in 2005 and 3.1 cases/million in 2006 (*17*).

The reasons for the increased incidence remain unclear. The incidence of listeriosis in France decreased substantially from 1987 through 1997 because of control measures implemented by the food industry in response to several large outbreaks (*15*). After mandatory reporting was implemented, incidence further declined from 4.5 cases/million persons during 1999–2000 to ≈3.5 cases/million persons during 2001–2003. As this reduction concerned all population groups (regardless of whether they were target groups for food recommendations), this further decline was essentially attributed to a reduction in exposure to contaminated food products (*15*). We are not aware of any changes in the control measures used by the food industry that could have increased exposure to contaminated foods in 2006 and 2007. However, in spite of these control measures, we cannot rule out that common food stuffs have been more frequently or more heavily contaminated with *L. monocytogenes* in the past 2 years (2006–2007).

Concerned by the large amount of disease attributable to hypertension-related conditions, in 2002, the French Food Safety Agency recommended a 20% reduction in average salt intake spread over 5 years (*18*). Consequently, the food industry reduced the salt content of selected products, such as ready-to-eat meat products. Evidence from routine food safety investigations indicates that a substantial proportion of ready-to-eat products, such as meat and fish products, may be contaminated by *L. monocytogenes* (*15*). The recently reduced salt content in some of these products, if contaminated, may have contributed to the growth of the organism and increased the likelihood of infection when the products were consumed by susceptible persons. To verify this hypothesis, surveys to determine not only the frequency but also the level of contamination by *L. monocytogenes* of these ready-to-eat food stuffs were initiated in 2008.

In France, we also observed an absolute and relative increase in patients with hematologic malignancies. Improved treatment has likely resulted in an increased number of patients surviving longer with these malignancies. Nevertheless, we are not aware of a sudden and recent increase in the number of these patients that could explain the upsurge. Further investigations are needed to assess whether changes in treatments for these patients may have contributed to an increased susceptibility for illness.

## Conclusion

The epidemiology of listeriosis in Europe is changing; the incidence is increasing, and the distribution of cases is shifting, primarily affecting elderly persons and those with predisposing medical conditions. The absence of large outbreaks suggests that there may be increasing exposure to foods that have sporadic or low-level *Listeria* contamination and that have some ability to support growth of *Listeria* organisms. The relative contributions of host and environmental factors need further study.
